# Artesunate suppresses the viability and mobility of prostate cancer cells through UCA1, the sponge of miR-184

**DOI:** 10.18632/oncotarget.15353

**Published:** 2017-02-16

**Authors:** Yan Zhou, Xiuju Wang, Jianjun Zhang, Aina He, Ya Ling Wang, Kun Han, Yang Su, Junyi Yin, Xiaobin Lv, Haiyan Hu

**Affiliations:** ^1^ Oncology Department of Shanghai Jiao Tong University Affiliated Sixth People's Hospital of Shanghai, Shanghai, China, 200233; ^2^ Key Laboratory of Malignant Tumor Gene Regulation and Target Therapy of Guangdong Higher Education Institutes, Sun Yat-Sen Memorial Hospital, Sun Yat-Sen University, Guangzhou, Yuexiu District of Guangzhou City, Guangdong Province, China, 510282; ^3^ Central Laboratory of the Third Affiliated Hospital, Nanchang University, Donghu District, Nanchang City, Jiangxi Province, China, 330008

**Keywords:** artesunate, prostate cancer, metastasis, UCA1, miR-184

## Abstract

Artesunate (ART) is a sesquiterpene lactone isolated from the leafy portions of the Chinese herb *Artemisia annua*. Here, we evaluated the effect of ART on the prostate cancer (PCa) cell lines DU145 and LNCaP and explored its potential mechanisms. ART inhibited the viability and mobility of DU145 and LNCaP cells. Mechanistically, we found that UCA1, one of the most important lncRNAs in malignancies of the urinary system, may be a potential mediator contributing to the tumor suppressor function of ART. First, the UCA1 level was reduced significantly after being exposed to ART. In addition, UCA1 was up-regulated in prostate cancer tissues compared to hyperplastic prostatic tissues, and a higher UCA1 level predicted poor prognosis in PCa patients. Furthermore, reintroduction of UCA1 into PCa cells reversed the effect of ART on apoptosis and metastatic ability. Then we determined that the miR-184/Bcl-2 axis might be the downstream signaling pathway of UCA1 upon ART treatment. UCA1 binds to miR-184 through its seed sequences and may function as a sponge for miR-184.

## INTRODUCTION

In the Western world, prostate cancer (PCa) is one of the main causes of cancer-related death and morbidity in men, especially in the elderly [1]. As a hormonally driven cancer, androgen deprivation therapy (ADT) either by castration or a chemical method is the preferred treatment strategy for low- and intermediate-risk PCa patients [2–5]. However, over 20% of patients are initially diagnosed with aggressive or treatment-refractory PCa. Moreover, most ADT-sensitive patients experience recurrence or metastasis, which eventually evolves into castration-resistant prostate cancer (CRPC) [6]. Over the last two decades, more and more effective inhibitors of the androgen-androgen receptor axis have been produced. However, most of these inhibitors simply prolong the time of progression to CRPC [7–9], making it important to explore new strategies. Traditional Chinese medicine is a promising option for the treatment of elderly tumor patients with poor KPS scores.

Artemisinin was extracted from *Artemisia annua* in the 1970s. Unlike most other antimalarials, artemisinin has a peroxide grouping but lacks the nitrogen-containing heterocyclic ring system. Although there are many side effects, such as lowered body temperature, analgesia, muscle relaxation, tremor, convulsions and lowered blood pressure, artemisinin has been widely used for its marked effect on malaria [10]. Then, the anti-tumor effect of artemisinin captured the attention of researchers. More derivatives with high-curative effects and low side effects were developed [11]. Artesunate (ART) is semi-synthetic water-soluble derivative of artemisinin, which was initially used as an effective antimalarial. ART contains a sesquiterpene lactone, which produces dramatically more cytotoxicity than artemisinin and converts to the active metabolite dihydroartemisinin (DHA) *in vivo* rapidly. Since the 1980s, ART has been tested for cytotoxicity on many kinds of tumors [12]. However, few researchers have studied the effect of ART on PCa. In addition, the molecular mechanism of the effects of ART on tumor cells also needs further exploration. Here, we verify that ART inhibited the proliferation and migration of PCa cells and explore the mechanism. We conducted a high-throughput screen of lncRNA changes in DU145 cells treated with or without ART. UCA1, first cloned from bladder cancer cells and shown to play an important regulatory role in malignancies in the urinary system [13], was identified as a key factor in the effect of ART on PCa cell lines. In addition, we provide evidence that UCA1 was up-regulated in prostate cancer tissues compared to hyperplastic prostatic tissues, and a higher UCA1 level predicted a poor prognosis in PCa patients.

## RESULTS

### ART repressed the viability and mobility of PCa cells *in vitro*

**Figure 1:**
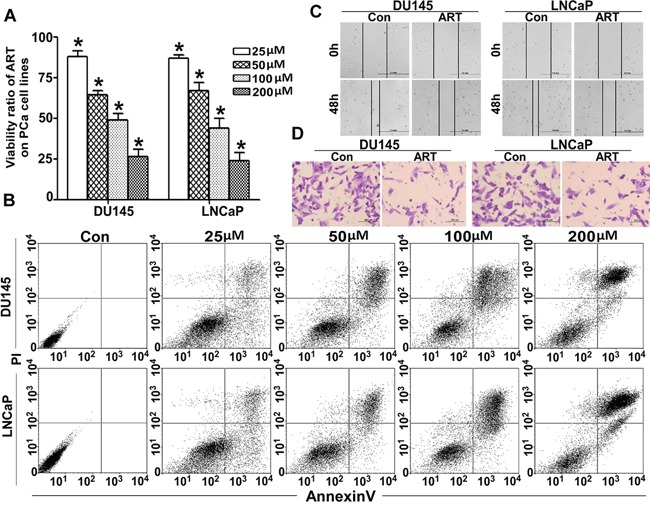
ART regulates cell viability and mobility in PCa cells **A**. After exposure to various concentrations (from 25 to 200 μM) of ART for 24 h, the viability of DU145 and LNCaP cells was assessed with a CCK-8 assay. The proliferation ratio decreased in a dose-dependent manner (*P < 0.001 compared with the untreated control group, which was considered 100%). **B**. Under identical culture conditions described in A, DU145 and LNCaP cells were also treated as described above. We collected both attached cells and those in suspension and calculated the apoptosis ratio with an Annexin V/PI FCM assay. The apoptosis ratio also increased in a dose-dependent manner. After incubation with IC50 of ART, the migration ability of DU145 and LNCaP cells was detected with a wound healing assay and a Transwell assay. **C**. The wound healing assay shows that ART impaired the wound healing; results were almost half of the control group. **D**. The migration rates of PCa cell lines decreased sharply in the ART group.

The ART inhibitory activity on PCa cells was assessed using a CCK8 kit assay. Dose-dependent experiments (from 25 to 200 μM) revealed that ART significantly inhibited the proliferation of both DU145 and LNCaP cells. The IC50 was established at 85.59 μM and 81.32 μM for DU145 and LNCaP cells, respectively. The cytotoxicity of ART was also verified with an Annexin V/PI staining apoptosis assay. Figure 1B shows that ART significantly induced apoptosis in a dose-dependent manner. ART not only inhibited the proliferation but also significantly reduced the invasiveness of PCa cells. To assess the anti-metastatic activity of ART, wound healing analysis and migration assays were performed. Figure 1C shows that, compared to the control group, the percentage of the wound closure in the DU145 and LNCaP cells treated with IC50 of ART was markedly decreased, with 65.6% vs 29.2% and 78.1% vs 42.7%. In a Transwell assay, ART dramatically inhibited the number of PCa cells that migrated through the Transwell membrane compared to the control group, as shown in Figure 1D.

### ART down-regulated UCA1 expression

**Figure 2:**
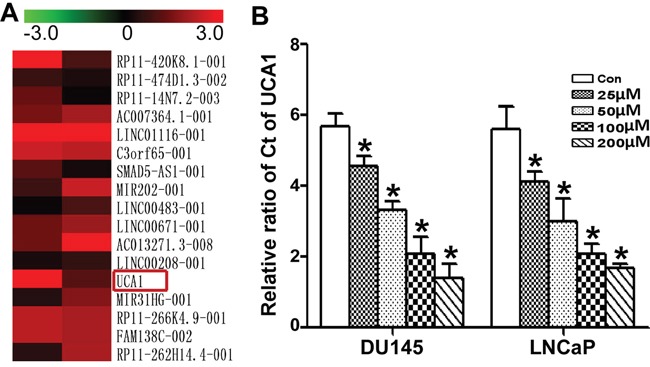
Comparison of the change in lncRNAs in PCa with ATR treatment **A**. The heat map shows part of lncRNA profile comparison in DU145 cells between the untreated group and the IC50 ART group. The most differentially down-expressed lncRNA was UCA1. **B**. After treatment with gradient concentrations (from 25 to 200 μM) of ART for 24 h, the UCA1 level in DU145 and LNCaP cells was measured by qRT-PCR. The valid qRT-PCR results indicated that ART inhibited UCA1 expression in a dose-dependent manner, which correlated well with the microarray data.

lncRNAs are involved in almost all types of cell biological processes. To explore the role of lncRNAs in the activity of ART, we screened the functional lncRNAs in DU145 exposed to IC50 of ART using a lncRNA microarray (Figure 2A). Among the changed lncRNAs, we focused on UCA1 because it is a cancer-related lncRNA. We also found that the level of UCA1 in DU145 and LNCaP cells was reduced in a dose-dependent manner (Figure 2B).

### UCA1 was up-regulated, and a higher UCA1 level was correlated with poor prognosis in PCa patients

**Figure 3:**
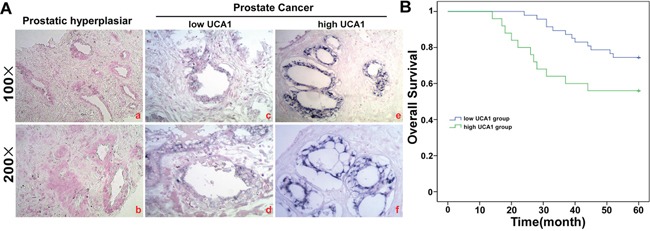
UCA1 is over-expressed in PCa tissue **A**. ISH analysis of UCA1 in the 72 cases of PCa tissue and 30 cases of hyperplastic prostate tissue using qRT-PCR. The difference is P = 0.000. **B**. Kaplan-Meier survival analysis of 67 EC patients, comparison is based on high and low miR-301 expression groups (P = 0.025).

To uncover the role of UCA1 in PCa, we compared the UCA1 level in PCa and hyperplastic prostate tissue sections by ISH. In the hyperplastic prostate tissue sections, there were no blue particles in the cell plasma. In contrast, positive particles were dispersed in the tubular gland tumor cells rather than mesenchymal tissues (Figure 3A). The mean age of PCa patients was 66.19 ± 9.35, ranging from 44 to 81. According to the median fold change of the ISH score of UCA1, which was 9.47, the PCa patients were divided into two groups, a low and a high expression group (n = 47 6.81 ± 1.86 vs n = 25 14.48 ± 2.58). The clinical characteristics of these two groups are shown in Table 1. High UCA1 expression was observed to be closely correlated with distant metastasis (P = 0.025), extracapsular extension (P = 0.010), advanced TNM stage (P = 0.009) and Gleason score (P = 0.030). In contrast, there was no correlation between UCA1 expression and other clinical factors, including age, PSA and seminal vesicle invasion (all P > 0.05). The correlation of UCA1 level and the overall survival of PCa patients was assessed using the Kaplan-Meier method. Patients in the high-UCA1 group had a significantly poorer prognosis than patients in the low-UCA1 group (74.5% vs 56%, P = 0.045; Figure 3B).

**Table 1:** The relationship between UCA1 expression and clinicopathological characteristics in PCa (n = 72)

Patient Characteristics		Low UCA1 group	High UCA1 group	P value
Age	<65	31	14	0.413
	≥65	16	11	
Distant metastasis	negative	42	16	0.025
	positive	5	9	
Extracapsular extension	negative	42	17	0.010
	positive	4	8	
Seminal vesicle invasion	negative	45	21	0.088
	positive	2	4	
Stage	I	10	2	0.009
	II	30	13	
	III/IV	7	10	
PSA level	<4	5	2	0. 663
	4-10	16	8	
	>10	26	15	
Gleason score	2-6	6	1	0.030
	7	25	13	
	8-10	16	11	

### UCA1 complementary sequence binds to miR-184 as ceRNA

**Figure 4:**
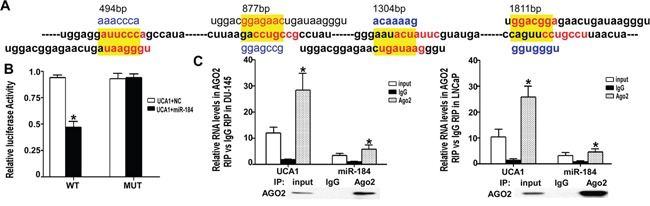
UCA1 regulated miR-184 as a ceRNA **A**. There were 4 binding sites between UCA1 and miR-184. The WT and mutated sequence are highlighted in red and yellow, respectively. **B**. The luciferase activity of WT was significantly decreased compared with NC, but for the MUT group there were no significant differences with NC (* indicates P < 0.05). **C**. After over-expression of UCA1 for 24 h, the total protein of DU145 and LNCaP cells was extracted and incubated with IgG or AGO2 antibody. Then, qRT-PCR was performed to analyze the relative levels of UCA1 and miR-184. For the AGO2 group, the UCA1 and miR-184 levels were significantly higher than in the IgG group (* indicates vs IgG group, P < 0.05).

To explore the contribution of the downstream signaling pathway of UCA1 to the tumor suppressor function of ATR, we identified the potential sponge function of UCA1 on miRNAs. miR-184 is a well-known tumor suppressor in a variety of cancers, and its seed sequence was identified as complementary to the UCA1 mRNA sequence at multiple sites (Figure 4A). A dual-luciferase reporter assay was used to investigate the functional interaction of miR-184 with UCA1. The binding sites and mutated fragments between miR-184 and UCA1 were inserted in a psiCheck2 vector downstream of the luciferase gene. The results of the luciferase assay (Figure 4B) indicated a potential interaction between UCA1 and miR-184. RNA-induced silencing complexes (RISCs) are formed when miRNAs combine with targets or lncRNAs. Ago2 is a key component of RISCs. Therefore, we performed a RIP assay using anti-Ago2 antibody on the extraction of UCA1 over-expressing DU145 and LNCaP cells. Figure 4C shows that UCA1 and miR-184 were significantly higher in Ago2-containing miRNAs than in the control IgG immunoprecipitates.

### UCA1 inhibits miR-184 expression and up-regulates the target genes of miR-184

**Figure 5:**
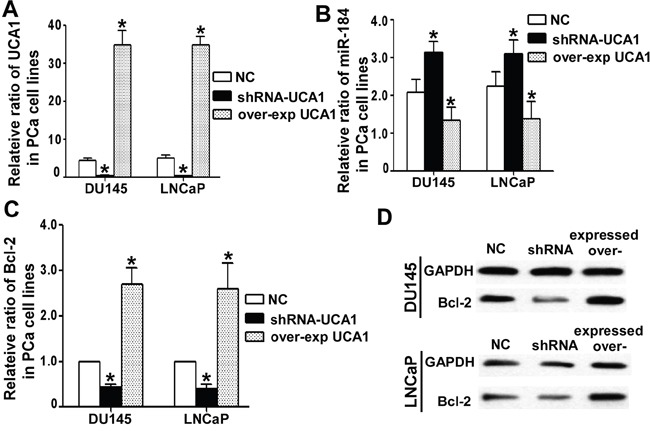
UCA1 regulated miR-184 and its target **A**. After over-expression and knock down of UCA1 for 24 h in DU145 and LNCaP cells, we detected the UCA1 level with qRT-PCR, and the over-expression group was more than 30, correspondingly the shRNA group was less than 0.5 (* indicates P < 0.05). **B**. UCA1 influenced the miR-184 level in DU145 and LNCaP cells by qRT-PCR. The higher the UCA1 level was, the lower the miR-184 level, and vice versa. (* indicates P < 0.05). For the Bcl-2 level, the tendency was the opposite of miR-184. UCA1 indirectly up-regulated the expression of Bcl-2 at both the mRNA **C**. and protein **D**. levels.

Based on the observation that UCA1 functionally interacts with miR-184 in PCa cells, we determined whether UCA1 affects the expression of miR-184 and its target genes. Bcl-2, a key anti-apoptotic factor and one of the verified direct targets of miR-184, [16] was chosen to test the UCA1 effect on miR-184. Overexpression or silencing of UCA1 in DU145 or LNCaP cells was accomplished with pcDNA3.1-UCA1 or pLK0.1 shRNA-UCA1 plasmids (Figure 5A). The expression level of miR-184 and Bcl-2 was evaluated using qRT-PCR. We found that the miR-184 expression level was inversely correlated with the UCA1 expression level (Figure 5B). However, in regard to its target Bcl-2, both the mRNA (Figure 5C) and protein level (Figure 5D) in DU145 and LNCaP cells exhibited the same tendencies as UCA1.

### Over-expression of UCA1 reversed the ART effect

**Figure 6:**
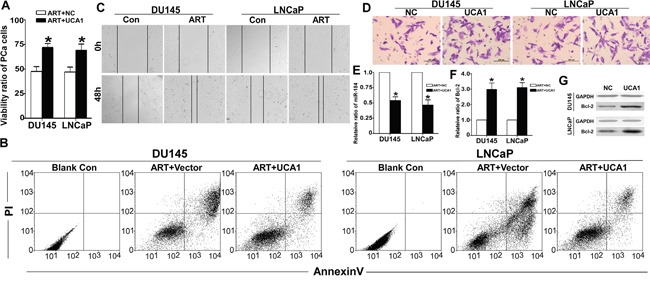
UCA1 is involved in the effect of ART on PCa cells After over-expression of UCA1 for 24 h, DU145 and LNCaP cells were treated with IC50 of ART for 24 h. **A**. Using a CCK8 assay, the viability ratio of the UCA1 over-expression group was dramatically higher than that in the NC group (* indicates P < 0.05). **B**. The apoptosis assay indicated that over-expression of UCA1 inhibited the apoptosis effect induced by ART. **C**. For the UCA1 group, the wound area was not healed than NC group. **D**. The Transwell assay demonstrates that UCA1 significantly promoted cell migration compared with the NC group. **E**. UCA1 also decreased the miR-184 level in DU145 and LNCaP cells by qRT-PCR. For the Bcl-2 level, the tendency was opposite to miR-184. UCA1 up-regulated the expression of Bcl-2 at both the mRNA **F**. and protein **G**. level.

To further verify whether UCA1 is a key mediator of ART, we over-expressed UCA1 in PCa cells upon ART-treatment. As expected, the viability ratio of both PCa cell lines significantly increased in the UCA1 over-expression group compared to the empty plasmid control group (Figure 6A). Consistently, the apoptosis ratio decreased compared to the control (Figure 6B). In addition, the wound healing analysis and migration assay indicated that over-expression of UCA1 reversed the migratory repression of DU145 and LNCaP cells upon ART treatment (Figure 6C and 6D). Furthermore, the increase of miR-184 in DU145 and LNCaP cells after exposure to ART was counteracted by over-expression of UCA1 (Figure 6E). For Bcl-2, the UCA1 over-expression group was much higher than the control group at both the mRNA and protein levels (Figure 6F and 6D).

## DISCUSSION

In the past decades, more and more natural and synthetic compounds have been identified as anticancer drugs, such as vinca alkaloids, arsenic trioxide, taxanes and others. Moreover, intensive study has uncovered new functions in older drugs. ART (ART) is a semisynthetic derivative of artemisinin. This compound first garnered the attention of researchers because of the improved pharmacological features in the treatment of malaria. Even more compelling, ART has fairly strong anticancer activity with few and insignificant side effects, and it is well-tolerated. The antileukemic effect of ART was the first and strongest anti-cancer action reported [17]. Here, we described the dose-dependent antitumor activity of ART in PCa cell lines through cell viability assays and an Annexin V/PI apoptosis assay. The IC50 values for ART in DU145 and LNCaP cells were 85.59 μM and 81.32 μM respectively. ART not only promoted apoptosis but also significantly suppressed the migration of tumor cells [18–20]. Bone is not a common site of metastasis for advanced PCa. Metastasis-free survival of PCa at 3 years is almost 50% [21]. Bone metastases not only induced pain but also led to the relevant risk of skeletal-related events [22]. When tumor cells acquire the capacity to migrate, there is a high risk of metastasis. The powerful migratory behavior is the foundation for tumor metastasis. Just as with the other studies, we also confirmed that ART prevented healing in a scraped wound assay and suppressed PCa cell migration through the membrane from the upper layer to the bottom of the aperture in a Transwell assay. Recent studies have shown that ART acts on the electron transport chain and is directly involved in reactive oxygen species generation, which leads to mitochondrial membrane depolarization and an apoptosis response cascade. Although many studies have led to great advances in our understanding of the mechanism of artemisinin, the molecular action of ART toward tumor cells is still unexplored. However, the details of the molecular mechanism remain unclear. In this report, we performed an lncRNA microarray. Throughout the entire genome, a vast majority of DNA segments are transcribed into non-coding RNAs. Based on their length, they are divided into miRNAs, approximately 20 nucleotides, and lncRNAs, longer than 200 bp, both of which are involved in post-transcriptional control and regulation of various biological functions. At present, we sought to define the lncRNA change in PCa cells exposed to ART using lncRNA-wide expression profiling. This is the first time that the role of lncRNA in the effect of ART has been discussed. The differentially expressed lncRNA identified as UCA1 is the most dramatically down-regulated (from 4.22 to 0.71) lncRNA. UCA1, located at 19p13.1, was first cloned in bladder carcinoma with a full-length of 1442 bp [23]. UCA1 is widely considered an oncogene, and it regulates cell growth and invasion in many types of tumors. Huang reported that UCA1 competitively inhibits the suppressive effect of hnRNP on p27, which significantly promotes breast cancer proliferation [24]. Fan reported that UCA1 plays an important role in the chemoresistance of bladder cancer by activating Wnt signaling in a Wnt6-dependent manner. Yang reported that UCA1 takes part in the PI3K pathway to regulate the cell cycle [25]. Cheng's results indicated that UCA1 is a key factor in the Akt/mTOR signaling pathway [26]. Aberrant over-expression of UCA1 has been found in melanoma [27], ovarian cancer [28], gastric cancer [29] and other malignancies. [30]. Our study presents the level of UCA1 in PCa and prostatic hyperplasia using an ISH method. For prostatic hyperplasia, it is negative in the tissue, without any blue particles. Correspondingly, blue positive granules were diffused in the plasma of PCa cells. We evaluated the UCA1 level using the ISH score, and the median fold-change was the cut-off value to divide the 72 PCa patients into low and high UC1 groups. The correlation between the UCA1 level and the clinical features of PCa patients revealed that advanced pathological stage (I/II, *n* = 25) and NSCLC lymph-node metastasis were significantly associated with UCA1 expression. In contrast, UCA1 expression was not associated with age, PSA and seminal vesicle invasion. The overall survival analysis was performed using Kaplan-Meier survival analysis and log-rank tests to further evaluate the correlation between the UCA1 level and PCa patient prognosis. High UCA1-expressing patients had a significantly lower survival ratio compared to low UCA1-expressing patients. Furthermore, how UCA1 works was further clarified. lncRNA may exert its regulatory function at the post-transcriptional level as a ceRNA to regulate miRs molecules. Bian [31] described that UCA1 works as a ceRNA to regulate the expression of miR-204-5p, inducing resistance to 5-FU in CRC. Thus far, miR-485-5p [32], miR-16 [33] and miR-216b [34] have also been shown to be regulated by UCA1 through a molecular sponge mechanism. Here, we found that there were four binding sites between miR-184 and UCA1. Therefore, we assumed that UCA1 also regulates miR-184 as a ceRNA. The luciferase assay verified our hypothesis. In different tumor types, miRs have different profiles and different effects. Yu described that the ectopic expression of miR-184 can indirectly suppress SHIP2 levels by interfering with the activity of miR-205, which triggered apoptosis in corneal epithelial cells [35]. Feng found that miR-184 suppressed proliferation and invasion in glioma and breast cancer cells [36]. Its anticancer function has also been verified in neuroblastoma [37]. However, in squamous cell carcinoma of the tongue [38] miR-184 works as an oncogene. Schaefer [39] detected the expression of 470 human miRNAs in twenty-four PCa tissue pairs by microarray and qRT-PCR, and miR-184 is one of the ten significantly down-regulated miRs in PCa. Here, we reported that UCA1 works as a ceRNA of miR-184. Both the luciferase assay and RIP results directly demonstrate UCA1 binding with miR-184. Furthermore, we also verified that miR-184 and its target Bcl-2 change according to the UCA1 level.

In summary, our study is the first demonstration that lncRNA participates in the antitumor effect of ART. ART significantly inhibited the viability and mobility of PCa cell lines triggered by UCA1 down-regulation. All the described clinical data support the proposition that over-expression of UCA1 may play a key role in PCa progression and development.

## MATERIALS AND METHODS

### Cell culture and cell viability detection

DU145 and LNCaP cells were cultured in DMEM with 10 % fetal bovine serum (Thermo Fisher Scientific, Inc. Waltham, MA, USA) at 37°C in a 5% CO_2_ humidified atmosphere. Cells were plated into 96-well plates with different concentrations of ART in 100 μL of medium for 24 h, and then, 10 μL of CCK8 reagent (Dojindo, Kumamoto, Japan) was added for 4 h according to the protocol. The cell viability was detected at 450 nm using a microplate reader (BioTek, Winooski, VT, USA). The formula for the cell viability ratio was as follows: (experimental group OD value/control group OD value) × 100%.

### Cell apoptosis assay

DU145 and LNCaP cells were cultured in 6-well plates with different concentrations of ART for 24 h. Both suspended and adherent cells were collected and stained with Annexin V-FITC and PI (BioVision, Palo Alto, CA) according to the manufacturer's instructions. Apoptosis and the cell death ratio were detected using a Beckman COULTER EPICS XL.

### Wound healing assay

In total, 2 × 10^6^ DU145 and LNCaP cells were grown to 80% confluence in 6-well plates. An artificial wound was scraped using a 200 μl pipette tip through the confluent monolayer. The wells were washed twice and incubated with fresh complete medium (DMEM) with or without half of the IC50 of ART. After 24 h, images were obtained to calculate the width of the wound using a microscope (objective ×4) (1×71, Olympus).

### Cell migration assay

Boyden chamber (Corning) assays were performed as Transwell assays. The upper chambers were coated with 50 μL of Matrigel (1:3 dilution; BD Biosciences, Franklin Lakes, NJ, USA) and 1×10^5^ cells were seeded in DMEM containing 1% fetal bovine serum. To the lower chambers, complete medium containing 10% fetal bovine serum was added. However, the concentrations of ART in both the upper and lower chambers were equal to those used in the wound healing assay. After incubating for 8 h, non-migrating cells on the upper layer were removed by scraping with a cotton swab. The cells that migrated through the filter were fixed with 4% paraformaldehyde for 15 min at room temperature and stained with 0.1% crystal violet solution for 20 min. The invading cells in five random fields were counted under 20× objectives and images were obtained.

### Microarray analysis of the expression of lncRNAs

Total RNA of DU145 cells treated with or without the IC50 of ART was isolated using TRIzol (Invitrogen). After quantification using a NanoDrop ND-2000 spectrophotometer, samples were hybridized to Agilent Human lncRNA (4 × 180K, Design ID: 042818) based on the manufacturer's standard protocols (Bohao Biocompany, Shanghai, China). After normalization with the log2 scale, the global expression of lncRNAs was mapped. We identified the differentially expressed lncRNAs with a discriminating parameter of q < 0.05.

### Tissue *in situ* hybridization for miRNA

Paraffin sections were obtained from 30 patients with prostatic hyperplasia and 72 patients who were diagnosed with PCa who were enrolled at the department of Oncology of Sun Yat-Sen Memorial Hospital and the Sixth People's Hospital of Shanghai from 2005 to 2010. All patients were followed by telephone or mail. Before the study began, we obtained the approval of the Ethics Committee of Sun Yat-Sen Memorial Hospital and the Sixth People's Hospital of Shanghai. The paraffin-embedded PCa and prostatic hyperplasia tissue sections were dewaxed with dimethylbenzene, dehydrated with graded ethanol, and washed 3 times with PBS. After digestion with 5 μg/ml proteinase K at 37°C for 5 min, the sections were pre-hybridized for 60 min at 37°C. Hybridization was performed with the UCA1 probe in the hybridization solution at 61°C for 18 h. After washing using SSC, sections were labeled with anti-digoxin IgG monoclonal antibody. The sections were then incubated with alkaline phosphatase-conjugated secondary antibodies for 2 h; the developing time of NBT/BCIP was approximately 15 min to detect UCA1 expression. Finally, the nuclei were counter-stained with nuclear fast red. Positive blue particles were observed in the plasma, and the score was evaluated. The evaluation criteria were determined as previously described [14].

### Bioinformatics analysis of the UCA1-miR-184 binding site

In StarBase, the most common database used for predicting a match in the seed region between lncRNAs and miR, we could not find a record of UCA1. Therefore, we designed software connecting the NCBI, UCSC RNAhybrid and Targetscan websites to explore lncRNA-miRNA, which set the complementary sites as more than 7 bp and the E value as more than 0.85. There were 34 miRs that had seed complementary binding regions with UCA1 (data not shown). We found that there were four binding sites between UCA1 and miR-184 and focused on those sites.

### Luciferase reporter assay

The fragment from UCA1 (406-2186 bp) containing all of the four binding seed sequences of miR-184 was amplified by PCR and recombined into psiCheck2 Dual-luciferase Target Expression Vector (Promega, Madison, WI, USA). The primer was forward 5′-**AACTCGAG**AGTGGCTGAAGACTGATGC-3′ (***XhoI*** site in bold) and reverse 5′-**AATGCGGCCGC**GACTGCCTTTGGGTTGAG-3′ (***NotI*** site in bold). The empty vector was used as the blank control (NC). HEK 293T cells were co-transfected with each psiCheck2 WT or MUT vector and with chemosynthetic miR-184 using Lipofectamine 2000. After 48 h, Renilla luciferase activities were measured consecutively using a dual-luciferase reporter assay system (Promega, Madison, WI, USA) following the manufacturer's instructions.

### RNA immunoprecipitation (RIP) assay

Based on a previous report [15], we performed a RIP assay to further verify the sponge function of UCA1 using an EZ-Magna RIP kit (Millipore, USA). DU145 and LNCaP cells with stable over-expression of UCA1 were collected and lysed with RIPA buffer to extract the protein. Then, the extract was incubated with RIP wash buffer which contains the A+G magnetic beads conjugated to human anti-Ago2 antibody (Millipore) or mouse immunoglobulin G (IgG). After the samples were digested with proteinase K, the immunoprecipitated RNA was isolated and UCA1 and miR-184 was detected using qRT-PCR.

### QRT-PCR assay

Total RNA was extracted using TRIzol, and the UCA1, miR-184 and Bcl-2 levels were evaluated using a PrimeScript miRNA RT-PCR Kit (Takara, Dalian, People's Republic of China) in accordance with the manufacturer's protocol. The primer pairs are shown in Table 2. The relative expression of UCA1 and Bcl-2 was calculated using the 2^−ΔΔCt^ method.

**Table 2:** The qRT-PCR primers

	forward	reverse
UCA1	5’-TTTGCCAGCCTCAGCTTAAT-3’	5’-TTGTCCCCATTTTCCATCAT-3’
Bcl-2	5’-ATGGGGTGAACTGGGGGAGGATTG-3’	5’-GGCCAGGCTGAGCAGGGTCTTC-3’
GAPDH	5′-ACAACTTTGGTATCGTGGAAGG-3′	5′-GCCATCACGCCACAGTTTC-3′

### Western blot assay

The cells were lysed in RIPA buffer and quantified with a BCA Protein Assay Reagent Kit (Thermo Fisher, USA). Briefly, 25 μg of the total protein was separated by SDS-PAGE and transferred onto PVDF membranes, and then, membranes were incubated with mouse monoclonal Bcl-2 antibody (1:1000, Abcam, USA) at 4°C overnight. After imaging with an enhanced chemiluminescence detection kit (ECL) (Thermo Fisher, USA), the band density was quantified using ImageJ lab software.

### Construction of the plasmids for UCA1 over-expression and silencing

The UCA1 segment, 2315 bp, was cloned from LNCaP cells and inserted into a pcDNA3.1 plasmid by Jierui Bio Com (Shanghai, China). The small interfering RNA sequence was 5’-accggtGTTAATCCAGGAGACAAAGATCAAGAGTCTTTGTCTCCTGGATTAACttttttgaattc-3 and was inserted into a pLK0.1 plasmid (Jierui Bio Com, Shanghai, China). After starving the cells in FBS-free DMEM for 24 h, the pcDNA3.1-UCA1 or pLK0.1 shRNA-UCA1 plasmids were separately transfected into DU145 or LNCaP cells using Lipofectamine 3000 reagent (Invitrogen, Carlsbad, CA, USA) according to the manufacturer's manual. After 6 h, the medium was changed to fresh complete medium.

### Statistical analysis

All data are described as the mean ± SD. SPSS version 18.0 software was used to analyze significant differences. One-way ANOVA was used to determine the significant differences. Differences were considered statistically significant when P was less than 0.05.
